# Adding a Prevention Intervention to an Established App: The Blued+ HIV Prevention Pilot Study in Beijing and Chengdu, China

**DOI:** 10.1002/jia2.70203

**Published:** 2026-09-15

**Authors:** Aaron J. Siegler, Guodong Mi, Fei Yu, Daniel Stegmueller, Kunru Ning, Shi Hao Ernest Koh, Yufen Liu, Kimberly A. Powers, Stefan Baral, Patrick S. Sullivan, Wenting Huang

**Affiliations:** ^1^ Department of Epidemiology, Rollins School of Public Health Emory University Atlanta Georgia USA; ^2^ Danlan Public Welfare Beijing China; ^3^ Department of Political Science Duke University Durham North Carolina USA; ^4^ National Center for AIDS/STD Control and Prevention, Chinese Center for Disease Control and Prevention Beijing China; ^5^ Department of Epidemiology, Gillings School of Global Public Health University of North Carolina at Chapel Hill Chapel Hill North Carolina USA; ^6^ Department of Epidemiology, Bloomberg School of Public Health Johns Hopkins University Baltimore Maryland USA

**Keywords:** digital intervention, HIV PrEP, pre‐exposure prophylaxis

## Abstract

**Introduction:**

HIV incidence among men who have sex with men (MSM) in China remains high, and the use of effective tools such as pre‐exposure prophylaxis (PrEP) is suboptimal. Smartphone app interventions are promising yet rarely move beyond the research sphere. Rather than inventing stand‐alone apps that rarely come to scale, integrating health interventions into existing multifunction apps would be a paradigm shift for research and practice.

**Methods:**

We conducted a prospective, single‐arm pilot study of an intervention platform built directly into Blued, a geosocial networking (dating) app for MSM with more than 6 million users in China. The intervention provided telePrEP care (including in‐app clinician services, reimbursed laboratory testing and mailed PrEP prescriptions), health messaging, and an order platform for home HIV tests and condoms. The pilot study, in Beijing and Chengdu, compared the intervention to contemporary repeated cross‐sectional comparison surveys. Primary outcomes were benchmarks for feasibility (≥30% home HIV test orders and ≥20% HIV PrEP prescriptions) and acceptability (≥71 System Usability Scale [SUS] score). Preliminary efficacy assessment compared changes in the intervention group to the repeated comparison groups with generalized estimating equations (GEE) models.

**Results:**

From July 2022 to December 2024, 423 people started the pilot intervention, 400 (95%) were retained at 6 months and 399 (94%) at 12 months. All primary outcomes at 12 months were significantly higher than prespecified benchmarks: 87% (366/423) ordered HIV tests, 42% (176/423) received PrEP prescriptions, and the mean SUS score was 73/100. In GEE models adjusting for group characteristics and the secular trend of the comparison groups, between baseline and 12 months, the intervention group had an absolute increase in PrEP use of 20% (95% CI: 13%–27%) and in HIV testing of 7% (95% CI: 1%–13%). There was no difference in condom use.

**Conclusions:**

In this study, we found that it is feasible and acceptable to incorporate HIV prevention interventions into existing apps. This model, with encouraging preliminary efficacy data, represents a new public‐private model for improving uptake of evidence‐based prevention interventions. Future research should test this model in a randomized clinical trial.

## 1 Introduction

Smartphone apps have become a central feature of most people's lives, with a global average of 5 h of app use per day in 2023 [1]. Given smartphone pervasiveness, technology‐based environments are promising for promoting health services. Many studies have capitalized on this; an umbrella review identified 48 meta‐analyses of app‐based health interventions [2]. The meta‐analyses identified many clinical trials with significant associations between app provision and positive preventive health outcomes. Despite this promise, few apps designed to promote preventive health have scaled beyond research to provide a population‐level impact. One review identified that despite considerable National Institutes of Health (NIH) investment in app research—more than 40 apps for HIV—no app was available for download in app stores for use outside of a research context as of 2021 [3]. This disconnect has been attributed to translation and download challenges [3]. Once research demonstrates an app has a positive health impact, a production model for rollout necessitates considerable resources—ongoing app maintenance requires developer time, and new developments in HIV prevention require researcher time. Translation, therefore, requires a scalable business with a revenue stream to offset ongoing costs, a challenging feat for researchers unaccustomed to business development. Once this exists, the target audience must be convinced to download the app; competition for downloads is highly competitive, with 1.96 million apps on the Apple App Store [4] and the average user having only 20 on their phone [3]. New approaches are needed to overcome these challenges to deliver on the promise of apps to improve preventive health.

Despite low HIV prevalence among general populations in China [5], HIV is highly prevalent among men who have sex with men (MSM), who comprise an estimated 50% of the population living with HIV [6]. High HIV prevalence is likely related to low levels of prevention services use: 53% of MSM in China reported no recent HIV testing [7], 40% reported not using condoms at last anal intercourse [8], and 89% reported never using pre‐exposure prophylaxis (PrEP) [9, 10]. Willingness to use PrEP among MSM in China is inversely associated with cost [11], perceived side effects [12], sexual minority‐related and HIV‐related stigma [13, 14], and lack of health insurance coverage [13]. To increase use of HIV prevention, developing interventions in the digital environments where MSM already spend time is a promising strategy.

We developed and piloted an electronic intervention to improve PrEP use, HIV testing, and condom use among Chinese MSM by working with a company that owns a widely used geosocial networking app for MSM. We collaboratively built the health platform into their app, Blued, for pilot testing. Blued has over 6 million monthly active MSM users in China, a figure that exceeds some estimates of the entire MSM population in the United States. The aim of the Blued+ pilot study, conducted in Beijing and Chengdu, was to determine the feasibility, acceptability and preliminary efficacy of adding a sexual health platform for MSM to an existing geosocial app.

## 2 Methods

The study protocol has been previously published [15]. This was a single‐arm pilot that compared outcome data from the intervention group to data from separately recruited participants that each completed a one‐time cross‐sectional survey (hereafter called “comparison group”). The study was funded by a call that did not allow for randomized designs. Cross‐sectional surveys were repeated over time to allow for adjustment for secular trends, enhancing rigour in study assessment in the absence of a randomized design. The study was registered at clinicaltrials.gov, NCT06647173.

Participants were recruited from Beijing and Chengdu using direct messages in the Blued app from July to August 2022. A brief screener survey determined study eligibility, which required (1) male sex at birth, (2) age ≥18 years, (3) anal sex with a man in the prior 6 months, (4) unknown or negative HIV status, and (5) eligible for PrEP according to China's consensus statement [16] by ever using PrEP/post‐exposure prophylaxis or any of the following in the prior 6 months—condomless sex, injection drugs or shared needles, an HIV positive sexual partner or a sexually transmitted disease diagnosis. Following informed consent, intervention participants had a 12‐month intervention period with surveys at baseline (month 0) and months 6 and 12. Cross‐sectional surveys for comparison group participants, also recruited from the Blued app and with identical eligibility criteria to intervention participants, were conducted at the same time as surveys for intervention participants to optimize comparability. Study activities were approved by the Emory University Institutional Review Board (IRB) (IRB00116983) and the Blued IRB. Study results are reported per CONSORT guidance for pilot trials [17].

The Blued+ intervention sought to change HIV prevention service use by lowering barriers to service access. Intervention development was informed by a prior evidence‐based digital intervention that increased PrEP use in the United States [18], three activity‐based focus group discussions (FGDs) conducted as part of the project in China in 2021 [15], and the framework of the Extended Unified Theory of Acceptance and Use of Technology (UTAUT2) [19, 20]. A table describing how UTAUT2 shaped intervention development is provided elsewhere [15]. Briefly, performance expectancy was operationalized as the provision of no‐cost prevention services in the home, effort expectancy led to a human‐centred interface with similarities to the already acceptable Blued platform, social influence was enacted through inclusion of not just health messages but also self‐care messages and habit formation was sought through multiple pathways to health action such as app navigation or clickable messages.

TelePrEP services consisted of in‐app study clinician PrEP counselling, reimbursed laboratory testing at local hospitals, in‐app clinician review of test results, prescribing of PrEP and mailing of 90‐day oral prescriptions with 200 mg emtricitabine (FTC) and 300 mg tenofovir disoproxil fumarate (TDF) tablets. We followed published Chinese guidance that required HIV, sexually transmitted infection (STI) and creatinine screening for all persons initiating PrEP; screening was conducted at local Chinese hospitals [16]. Translated versions of health messages from the prior intervention [18] were included in FGDs to inform their adaptation. During the intervention period, 29 messages were sent to all participants. Messages targeted PrEP use promotion (*n* = 11), condom and lubricant use (*n* = 6), HIV test promotion (*n* = 6), engagement in healthcare (*n* = 2), and health‐positive self‐care (*n* = 3). The health messages [14] featured direct calls to action such as links to the PrEP services and home HIV test ordering offered in the app. The in‐app online order platform allowed participants to receive a choice of home HIV tests (fingerstick blood, urine or oral fluid), choice of condoms (e.g. ultrathin or standard) and choice of lubricant (water‐based or silicone‐based) delivered to an address of their choosing, with re‐orders allowable each quarter. HIV counselling was available through the app for participants testing positive. Intervention participants received the prevention package within the Blued+ app as an update to their existing Blued app. More details on telePrEP provision and other intervention features are provided elsewhere [15]. To improve dissemination, a video walkthrough demonstrating intervention features within the app can be accessed online [21]. This allows for an understanding of the layout, feature set, and pathway through the intervention as experienced by participants. Such walk‐throughs facilitate transferability to other platforms and settings by improving interpretability of intervention features in the context of their provision [3].

Participants in the cross‐sectional comparison group could not access the intervention, but instead had access to existing sexual health services offered on the Blued platform through HeHealth. This included in‐person HIV testing and condom distribution scheduling or pharmacy‐based home HIV tests and condom ordering. After a PrEP prescription had been received from a local in‐person clinician, comparison participants could receive it through the online pharmacy. HeHealth is for‐profit and resembles an online pharmacy; unlike the intervention, it does not provide health messaging or guidance regarding prevention good choices and PrEP access.

Study outcome data came from PrEP prescription data from study providers, product order data from the study app and online self‐completed surveys conducted on the secure study platform [22]. To facilitate comparability, the intervention group and the repeated cross‐sectional comparison groups completed the same survey, except for a few additional questions relating to acceptability of the intervention. A full description of survey sources and items is available [15]. Sociodemographic items came from the Chinese census [23], the scales from previously validated instruments [24, 25, 26, 27], and sexual and prevention behaviours were adapted from the American Men's Internet Survey (AMIS), a repeated cross‐sectional survey [28].

The primary pilot study outcomes, proposed in 2019, were go/no‐go cutpoint criteria for whether the intervention should proceed to a future clinical trial [15]. Criteria were based on models of service uptake required to substantially impact an HIV epidemic and on published interpretations of the System Usability Scale (SUS); we used a validated Chinese version of the SUS [24]. The feasibility “go” rule was ≥30% of participants ordering and receiving home HIV tests and ≥20% ordering and receiving HIV PrEP prescriptions. The acceptability “go” rule was a mean intervention SUS score of good or higher (≥71 out of 100) [24, 25]. Primary study outcomes compared the intervention group to prespecified benchmarks with binomial tests (categorical outcomes) or *t*‐tests (continuous outcomes). Among intervention participants who initiated PrEP, a preliminary assessment of adherence was measured at 9‐month follow‐up. Participants visited a local laboratory for dried blood spot collection to measure tenofovir diphosphate (TFV‐DP) levels. We evaluated preliminary adherence for daily oral PrEP users with a cutpoint indicating adherence >4 doses/week (>700 fmol/punch TFV‐DP). For on‐demand PrEP users, we used the minimum detectable threshold for the test (>80 fmol/punch) as a cutpoint to indicate greater than zero PrEP use in the past 4−8 weeks.

Preliminary intervention efficacy was determined by comparing the intervention group to repeated cross‐sectional controls for the three domains the intervention targeted for change: HIV testing, condom use, and PrEP use, each measured with dichotomous (yes/no) self‐report data for the previous 3 months using survey items adapted from AMIS [28]. To gain an assessment of preliminary efficacy, we used growth models based on generalized estimating equations (GEE) to account for repeated measures within individuals over time. GEE logit models calculated the increase in the percentage of intervention group participants who reported prevention service (e.g., PrEP) use from baseline to endline, controlling for secular trends observed in the comparison groups. We used propensity scores to adjust for differences in baseline covariates between the intervention group and the comparison group because our study did not have a randomized design [29, 30, 31]. The weighting approach (inverse odds of selection weights) [30] used a vector of baseline covariates of age, income, employment, education, study site, PrEP awareness, ever PrEP use, HIV testing, and recent PrEP use. Models were not adjusted for their outcome because the outcome is itself a difference of post‐ and pre‐intervention levels that adjusts for baseline differences. Using covariates, we estimated the propensity score of intervention sample membership via logistic regression in the combined intervention and comparison sample, to calculate the inverse odds of sampling weights. This reweights the intervention sample to resemble baseline comparison population characteristics. We also explored specifications that restricted the sample to a common propensity score support region and that used full matching. Full details regarding the modelling strategy and alternative specifications are available in the Supplementary Appendix.

## 3 Results

**FIGURE 1:**
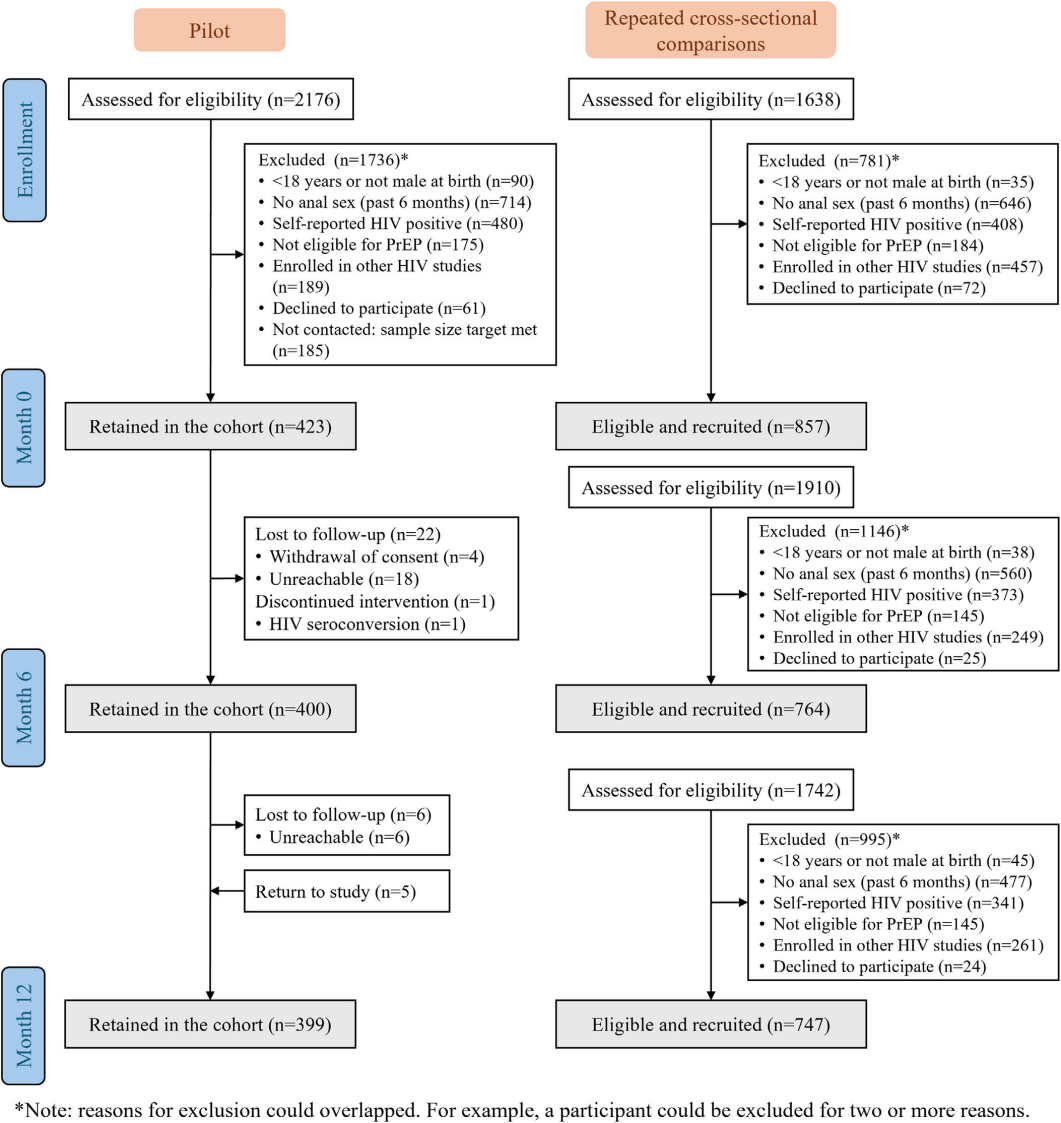
CONSORT flow diagram of participant enrolment and retention for Blued+ pilot study and comparison groups, Beijing and Chengdu, China, 2022−2023. All pilot participants received the Blued+ mobile app HIV prevention intervention that included HIV pre‐exposure prophylaxis (PrEP) provision through telemedicine within the app. Repeated cross‐sectional surveys were conducted for comparison purposes.

From July to August 2022, 2176 participants were assessed for eligibility, and 650 were eligible for enrolment (Figure 1). The pilot cohort target of 423 participants was enrolled from this group based on the order in which they had completed eligibility surveys. Retention rates for intervention participants were 95% (400/423) at 6‐month and 94% (399/423) at 12‐month post‐baseline. For the repeated cross‐sectional comparison groups, there were 857 participants at baseline, 764 participants at 6 months and 747 participants at 12 months.

Sociodemographic and sexual health variables for the intervention group and for each of the three repeated cross‐sectional cohort groups are displayed in Table 1. The groups were similar in age (mean = 30 years), college education (range: 53%–57%), full‐time employment (range: 78%–82%), identify as gay (range: 81%–86%), income (range: 41%–46% >$1400 USD) and ever use of PrEP (range: 21%–27%). Relative to the comparison groups, the intervention group at baseline had a lower percentage of Beijing residents (54% vs. 66%–69% range) and higher percentages reporting condom use at last sex (63% vs. 54%–59% range), HIV testing in the prior 3 months (71% vs. 59%–64% range) and ever having heard of PrEP (96% vs. 84%–91% range).

**TABLE 1:** Baseline demographics of Chinese MSM participating in a pilot study of a mobile app‐based HIV prevention service for intervention group and repeated cross‐sectional comparison groups at 0, 6 and 12 months, Beijing and Chengdu, 2022−2023.

	Intervention group	Repeated cross‐sectional comparison groups		
	Baseline (*n* = 423)	Month 0 (*n* = 857)	Month 6 (*n* = 764)	Month 12 (*n* = 747)
Age in years, mean±SD	30±7	31±8	31±7	30±7
City, *n* (%)				
Beijing	230 (54)	565 (66) ^**^	527 (69)	510 (68)
Chengdu	193 (46)	292 (34)	237 (31)	237 (31)
Education, *n* (%)				
Postgraduate and above	89 (21)	216 (25)	190 (25)	187 (25)
College or university	235 (56)	455 (53)	428 (56)	413 (55)
Associate's degree and/or technical school	84 (20)	137 (16)	115 (15)	111 (15)
High school and below	15 (4)	49 (6)	31 (4)	36 (5)
Employment status, *n* (%)				
Employed full‐time	328 (78)	675 (79)	626 (82)	583 (79)
Employed part‐time	31 (7)	38 (4)	41 (5)	38 (5)
Full‐time student	43 (10)	95 (11)	62 (8)	90 (12)
Part‐time student	2 (<1)	2 (<1)	3 (<1)	2 (<1)
Unemployed/retired	13 (3)	42 (5)	25 (3)	23 (3)
Others	6 (1)	5 (1)	7 (1)	11 (1)
Income (monthly, in USD), *n* (%)				
>1400	173 (41)	365 (43)	352 (46)	343 (46)
980−1400	96 (22)	205 (24)	189 (25)	169 (23)
420−979	95 (22)	185 (22)	161 (21)	154 (21)
< 420	59 (14)	102 (12)	62 (8)	81 (11)
Sexual orientation, *n* (%)				
Gay	357 (84)	696 (81)	639 (84)	643 (86)
Bisexual	65 (15)	155 (18)	122 (16)	96 (13)
Heterosexual and others	1 (<1)	6 (1)	3 (<171)	8 (1)
Condomless anal sex^a^, *n* (%)				
Yes	265 (63)	505 (59)	415 (54)	432 (58)
No	158 (37)	352 (41)	349 (46)	315 (42)
HIV testing^a^, *n* (%)				
Yes	302 (71)	501 (59) ^**^	461 (60)	476 (64)
No	121 (29)	356 (41)	303 (40)	271 (36)
Heard of PrEP, *n* (%)				
Yes	404 (96)	723 (84) ^**^	676 (89)	679 (90)
No	19 (4)	134 (16)	88 (11)	68 (9)
PrEP use, ever, *n* (%)				
Yes	114 (27)	182 (21) ^*^	166 (22)	203 (27)
No	309 (73)	675 (79)	598 (78)	544 (73)
PrEP use^a^, *n* (%)				
Yes	80 (19)	132 (15)	139 (18)	184 (25)
No	343 (81)	725 (85)	625 (82)	563 (75)

Abbreviations: MSM, men who have sex with men; PrEP, HIV pre‐exposure prophylaxis; SD, standard deviation.

^a^ In the past 3 months.

^*^ *p*<0.05, ^**^ *p*<0.01 for *t*‐test (for age) and chi‐square test between intervention group baseline and repeated cross‐sectional comparison group month 0.

The primary study outcomes for the intervention group significantly exceeded a priori benchmark criteria for intervention feasibility and acceptability (Table 2). For the feasibility assessment, the proportion of intervention participants ordering home HIV tests was 87% versus a benchmark of ≥30%, and the proportion of participants receiving PrEP prescriptions was 42% versus a benchmark of ≥20%. For the acceptability assessment, the mean SUS score was 73/100 versus a benchmark of ≥71/100. The SUS score was significantly higher among participants who only used daily PrEP (mean, SD: 81.7, 15.5) than among participants who only used on‐demand PrEP (mean, SD: 73.9, 14.4).

**TABLE 2:** Primary Blued+ pilot primary outcomes at 12 months follow‐up compared to pre‐specified benchmarks for feasibility and acceptability.

	Measure	Pre‐specified benchmark	Study outcome % (*n*/*N*) or mean±SD	*p*‐value^a^
**Feasibility**	Home HIV test orders	≥ 30%	87% (366/423)	<0.01
**Feasibility**	PrEP prescriptions received	≥ 20%	42% (176/423)	<0.01
**Acceptability**	System Usability Scale (SUS)	“Good” score (≥ 71/100)	73.9 ± 14.4	<0.01

Abbreviation: SD, standard deviation.

^a^ Significance tests compared study outcomes at 12 months to the pre‐specified benchmark level. Binomial tests were used for categorical outcomes (home HIV test orders and pre‐exposure prophylaxis [PrEP] prescriptions received), and a *t*‐test was used for continuous outcome data (System Usability Scale score).

This table presents a priori planned analyses to determine if a mobile HIV prevention app, Blued+, met sufficient feasibility and acceptability criteria in this pilot study to proceed to further clinical trial research.

**FIGURE 2:**
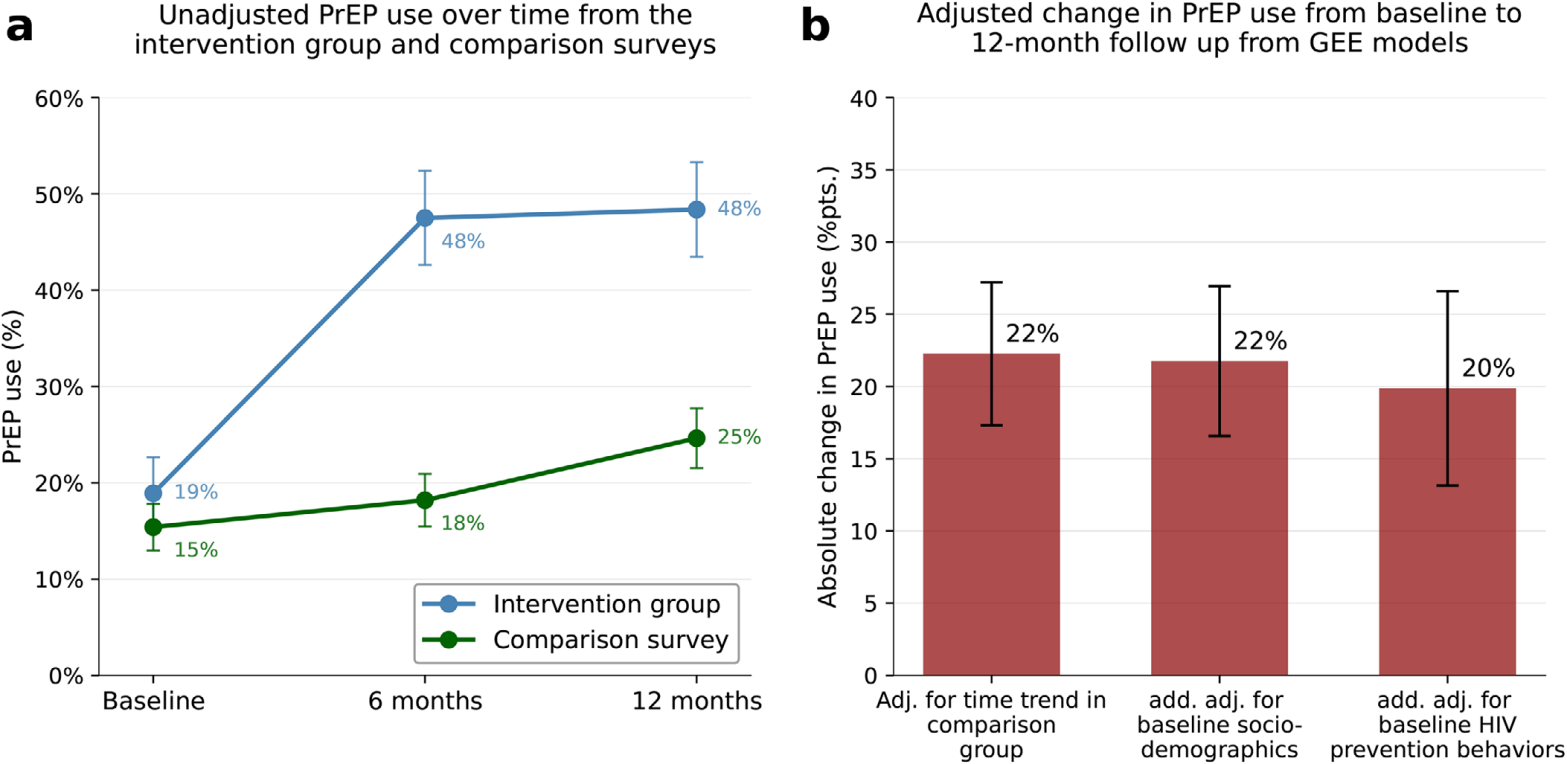
Growth model with intervention uptake relative to repeated cross‐sectional comparison groups over time for self‐reported PrEP use in the last 3 months. Panel (a) provides unadjusted estimates of HIV pre‐exposure prophylaxis (PrEP) use, and panel (b) provides generalized estimating equations (GEE) model estimates of PrEP use. In panel (b), the left GEE model is adjusted for the time trend in comparison groups. The centre model additionally adjusts for differences in baseline sociodemographic characteristics between the intervention group and cross‐sectional comparison groups. The right model additionally adjusts for baseline PrEP awareness and baseline HIV prevention behaviours.

**FIGURE 3:**
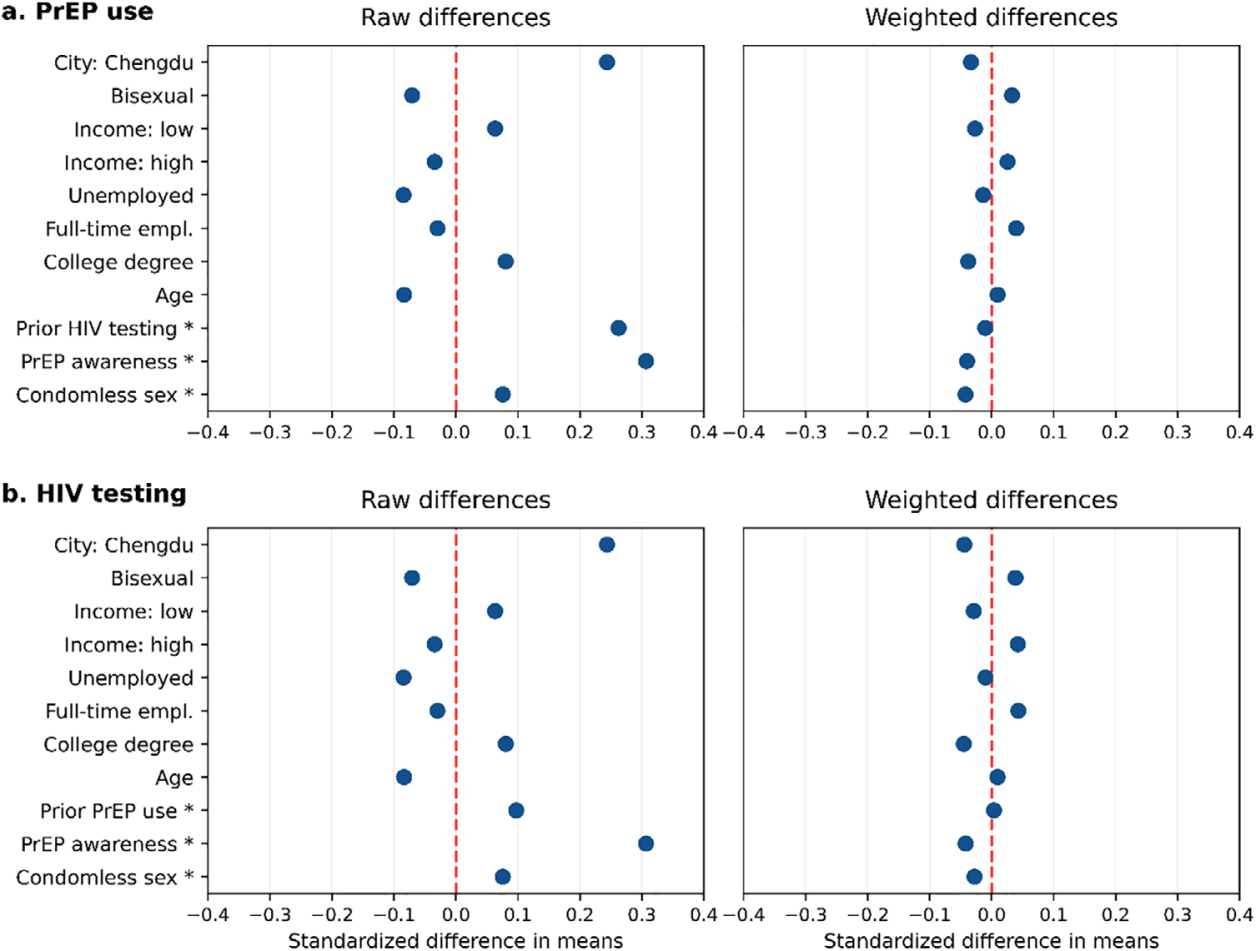
Raw and inverse probability weighted standardized mean differences between covariates for the Blued+ pilot intervention group versus the repeated cross‐sectional comparison group at baseline before and after applying inverse odds of sampling weights.

PrEP use in the prior 3 months in the intervention and comparison groups (respectively) was 19% (80/423) and 15% (132/857) at baseline, 48% (190/400) and 18% (139/764) at 6 months follow‐up, and 48% (193/399) and 25% (184/747) at 12 months follow‐up (Figure 2a). Longitudinal GEE models assessed changes in the intervention group from baseline to endline, adjusting for the observed secular trend in the comparison groups (Figure 2b, left graphic). The absolute increase in the proportion of intervention participants using PrEP was 22% (95% confidence interval [CI]: 17%–27%). In a model that additionally adjusted for baseline differences in sociodemographic characteristics (Figure 2b, centre graphic), the estimated absolute increase in PrEP was unchanged at 22%(95% CI: 17%–27%). When adjusting for additional baseline covariates (Figure 2b, right graphic), the estimated absolute increase in PrEP was 20%(95% CI: 13%–27%). The impact of inverse probability weighting on baseline covariates can be seen in Figure 3. Details of GEE models and adjusted comparison group covariates can be found in the Supplementary Appendix.

**FIGURE 4:**
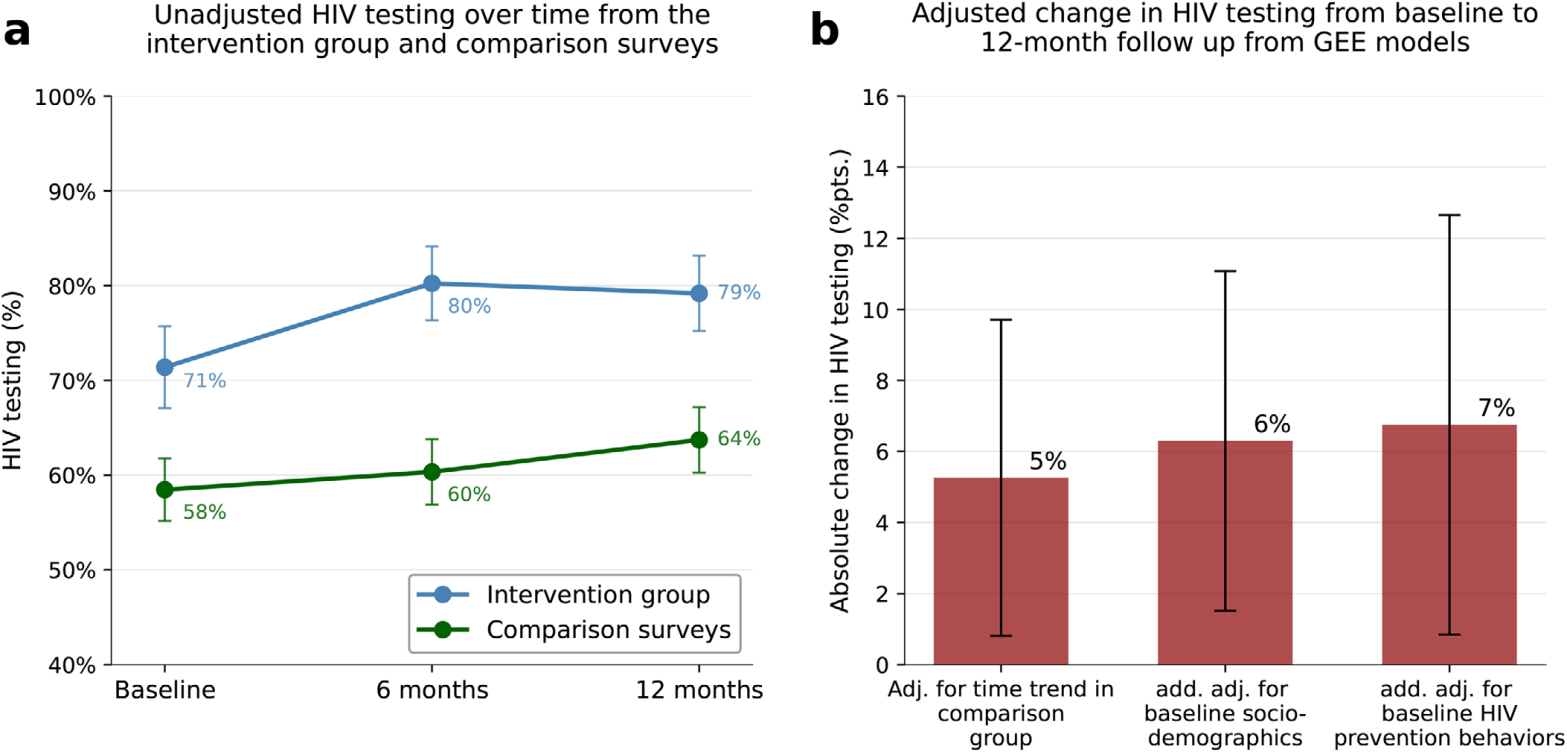
Growth model with intervention uptake relative to repeated cross‐sectional comparison groups over time for self‐reported HIV testing in the last 3 months. Panel (a) provides unadjusted estimates of any HIV testing in the last 3 months, and panel (b) provides generalized estimating equations (GEE) model estimates of any HIV testing in the period.

HIV testing in the prior 3 months in the intervention and comparison groups (respectively) was 71% (302/423) and 58% (501/857) at baseline, 80% (321/400) and 60% (461/764) at 6 months follow‐up, and 79% (316/399) and 64% (476/747) at 12 months follow‐up (Figure 4a). Longitudinal GEE models assessed changes in the intervention group from baseline to endline, adjusting for the observed secular trend in the comparison groups (Figure 4b). The absolute increase in the proportion of intervention participants testing for HIV ranges from 5% (95% CI: 1%–10%) to 7% (95% CI: 1%–13%) in the three models.

Condom use in the prior 3 months in the intervention and comparison groups were similar at each time point (respectively): 37% (158/423) and 41% (352/857) at baseline, 43% (173/400) and 46% (349/764) at 6 months follow‐up, and 43% (170/399) and 42% (315/747) at 12 months follow‐up. Longitudinal GEE models identified no changes from baseline to endline in condom use.

Preliminary adherence assessment among PrEP users in the intervention identified 7/14 (50%) of daily PrEP users had adherence ≥4 doses/week (TFV‐DP>700 fmol/punch). For on‐demand PrEP in the past 3 months, 42/69 (61%) had >0 PrEP use in the past 4−8 weeks (TFV‐DP above minimum detectable threshold, >80 fmol/punch).

**FIGURE 5:**
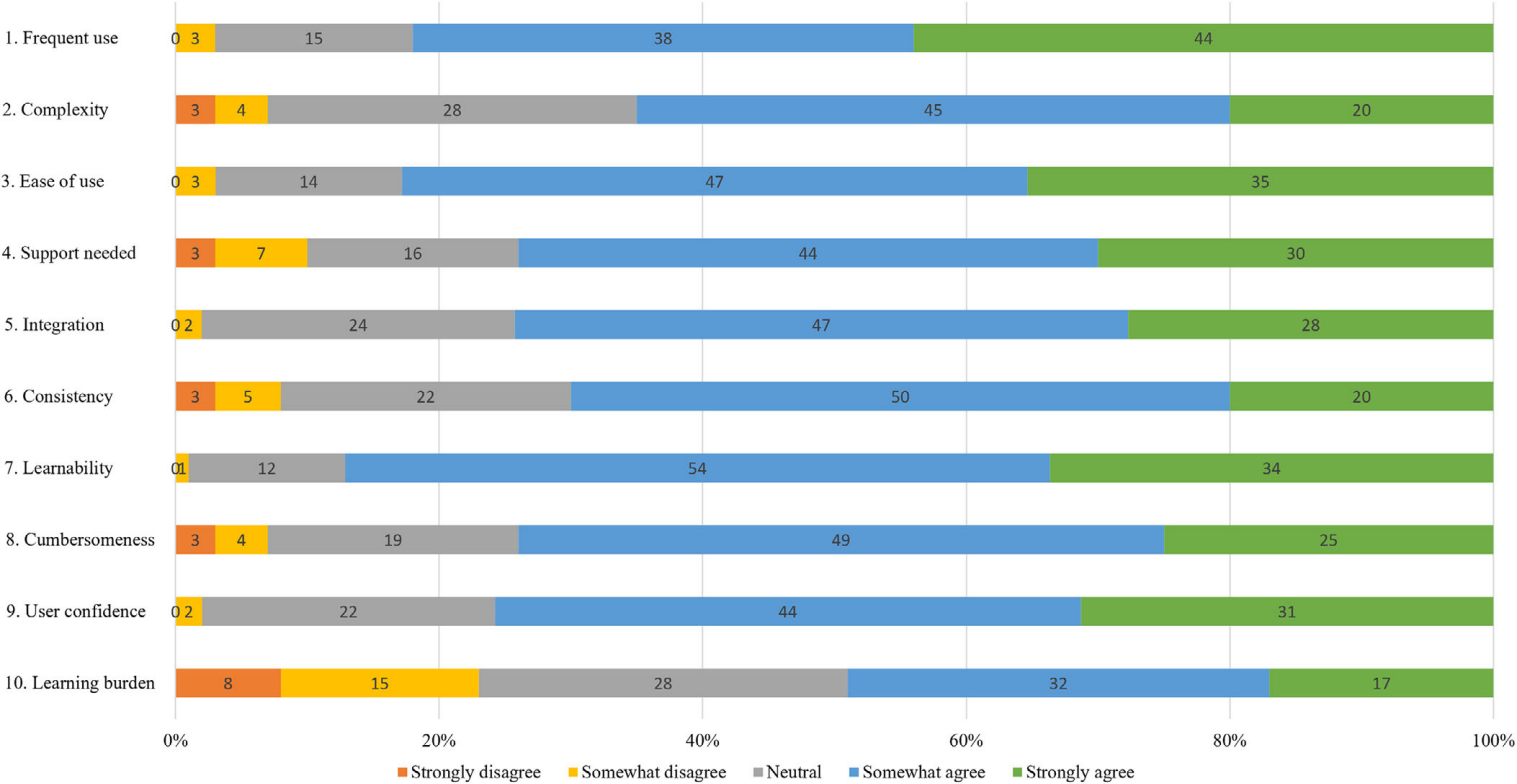
System Usability Score by items at month 12 follow‐up. Even‐numbered items (negative statements) are reverse‐coded. For all items, “strongly agree” represents a high level of intervention acceptance and “strongly disagree” represents a low level of acceptance. Statements in English and Chinese translations can be found in the Supplementary Appendix.

SUS item performance is shown in Figure 5, with negative items reverse‐coded to ease interpretability. All items except one had a substantial majority of participants “strongly agreeing” or “agreeing” in a positive direction. Items with the highest endorsement were speed of learning the Blued+ app and ease of use. One item had lower acceptability; about one‐quarter of participants rated that they needed to learn to use the app. It is possible this item had less positive ratings because the intervention facilitated access to PrEP, a concept not all participants were familiar with.

The intervention allowed participants to choose different home HIV test and condom/lubricant modalities, and this feature was substantially used. Overall, 87% (366/423) of participants ordered at least one type of home HIV test. Specifically, 14% (60/423) ordered a urine HIV test, 11% (48/423) ordered an oral fluid HIV test and 68% (288/423) ordered a blood‐based HIV test. Participants were able to order multiple times across the 12‐month study period, so order categories are not mutually exclusive. Overall, 89% (376/423) of participants ordered condoms and lubricants. A total of 76% (322/423) ordered premium brand condoms, 50% (210/423) ordered the ultrathin brand condoms and all participants who ordered any condom received standard condoms. Nearly all participants ordering lubricants opted to receive both water‐ and silicone‐based lubricants, 85% (359/423).

## 4 Discussion

This pilot study assessed the preliminary efficacy of Blued+, an HIV prevention intervention that included telemedicine PrEP provision, in Beijing and Chengdu, China. To our knowledge, this study is the first to integrate an HIV prevention platform directly into an existing, widely used app. We found high feasibility, acceptability and preliminary efficacy for this approach. The pilot intervention with over 400 users led to an absolute increase in PrEP use of 20 percentage points and in HIV testing of 7 percentage points compared to what would be expected based on the comparison group. In the context of low overall uptake of PrEP among Chinese MSM [9, 10], the large absolute increase in PrEP use is especially noteworthy.

The intervention had a “good” System Usability Score and substantially exceeded our prespecified feasibility and acceptability benchmarks for continuing this line of research. Our preliminary biomarker assessment of PrEP use among persons in the intervention group encouragingly found that a majority of persons prescribed PrEP had evidence of use during the period. The foundational premise of the intervention was that by bringing HIV prevention interventions into trusted electronic spaces, we could facilitate health‐positive choices. This study indicates the promise of the overall approach—increasing use of prevention services by meeting people where they live.

Using existing apps to reach people with offers of prevention services is scalable beyond this project, for instance with other geosocial networking companies. The formation of a partnership between a private business with an existing successful app and academic researchers was central—the business was needed to provide the existing platform and to build new services onto its platform, and the academic partner facilitated the use of an evidence‐based approach and rigorous intervention testing. Scale‐up of this model will require public health entities to supply prevention goods and funds to sustain intervention provision. The offline model of prevention good provision in many countries, including China, indicates this is feasible—public funding supports prevention good costs for condoms and HIV tests and dissemination of those goods by funding community organizations. In late 2025, some media coverage reported the shutdown of Blued. Blued is currently available from both major app stores in China, and an enhanced, dialogue‐based version of the app is available in international markets with functionality not substantially changed.

The prevention services in the app were highly used, with over 80% of intervention users ordering home HIV tests and 42% of users receiving PrEP prescriptions through the platform. Even though HIV tests and PrEP referrals are offered in‐person by local health departments in China, the online access provided by our intervention facilitated greater use of these services relative to cross‐sectional comparison groups. In studies assessing stand‐alone prevention apps in the United States, one found 13% of participants used the provided home HIV tests [32], and another found 53% of participants had ordered home HIV tests [33]; both levels were lower than those observed in this study that provided services from within an existing platform.

Condom orders were relatively high in this study, with nearly 9 out of 10 users ordering condoms and lubricant. Yet, this did not translate into differences in reported condom use in the intervention group, with no differences observed between baseline and endline condom use. Although condoms are highly effective and have low failure rates for anal sex [34], evidence for increasing condom provision is mixed: one review identified that condom provision interventions increased reported condom use [35], whereas another review identified a limited impact of condom provision interventions [36]. Further research is needed to explore reasons that provision of condoms does not consistently increase use.

This study has limitations. First, due to requirements of the grant mechanism, the pilot study was not randomized. We sought to address concerns about unrelated secular changes that could influence study findings by conducting repeated cross‐sectional comparison surveys at the same time as assessments were done for the intervention group. Second, the study was conducted in two cities—generalizability may be limited for areas with different healthcare settings. Third, our design did not collect some contextual data such as STI incidence. Fourth, the primary study outcome involved self‐reported use of prevention resources, presenting potential for misclassification and social desirability bias. Fifth, the preliminary assessment with biomarkers was limited regarding on‐demand PrEP; optimal on‐demand adherence assessment requires frequent surveys and a biomarker with a shorter cumulative drug adherence period. Notably, for the intervention group, orders of HIV prevention services for tests and PrEP medication provide some preliminary indication of validity to self‐report. A future randomized, controlled trial with appropriate biomarker outcomes will be needed to more firmly establish intervention effects.

## 5 Conclusions

We added HIV prevention services to a widely used app to test a new model of providing HIV service provision. The approach has the potential to overcome challenges faced by prior interventions that have demonstrated efficacy during research but have had limited real‐world impact due to issues relating to scale‐up [3]. Further assessment is needed; our next planned step is a clinical trial to measure intervention efficacy and cost‐effectiveness. In the long run, the intervention will require minimal but not insubstantial public investment, including the provision of prevention commodities and the costs of the platform. The results of this pilot study indicate the considerable promise of building interventions into existing digital spaces to increase service accessibility and scalability.

## Author Contributions

AJS drafted the manuscript. AJS and PSS conceived the study and obtained funding as the lead investigators. GM and YL oversaw study conduct in China as the site investigators. FY and KN were responsible for participant recruitment and data collection as site coordinators. WH and SHEK cleaned data and conducted baseline descriptive data analyses. DS conducted advanced analyses as the study statistician. All authors provided feedback and edited the manuscript.

## Funding

The study was supported by the US National Institute of Allergy and Infectious Diseases (R01AI181799).

## Conflicts of Interest

Emory University received funding from the National Institutes of Health (NIH) to support work on this study. Guodong Mi and Fei Yu work for Danlan Public Welfare, which is funded in part by Blued.

## Disclaimer

The funders of the study, the National Institute of Allergy and Infectious Diseases, US National Institutes of Health, had no role in study design, data collection, data analysis, data interpretation or writing of the report. The content is solely the responsibility of the authors and does not necessarily represent the official views of the National Institutes of Health.

## Supporting information




**Supporting File 1**: jia270203‐sup‐0001‐Appendix.docx.

**Supporting File 2**:jia270203‐sup‐0002‐Appendix1.pdf.

**Supporting File 3**:jia270203‐sup‐0003‐Appendix2.docx.

## Data Availability

The data that support the findings of this study are available from Beijing Centers for Disease Control and Prevention. Restrictions apply to the availability of these data, which were used under a data sharing agreement between Blued and Emory University for this study. Researchers can access the data by contacting Hongyan Lu at hongyanlu@chinaaids.cn. Lu was not involved in this research study.
