# Supplementary material for: Adding a Prevention Intervention to an Established App: The Blued+ HIV Prevention Pilot Study in Beijing and Chengdu, China
Source: J Int AIDS Soc. 2026 Sep 15;29(9):e70203. doi: 10.1002/jia2.70203 (PMC13575770; doi:10.1002/jia2.70203)
Supplement: Supplementary file 1 [file JIA2-29-e70203-s002.pdf]

Appendix

A.1 GEE model

Let y_{it} denote the observed binary outcome (PrEP use, HIV test) for individual i ($i = 1, \dots, 400$) in wave t ($t = 0, 1, 2$). D_{it} is an indicator variable for the post-intervention period equal to 1 if $t \geq 1$ and 0 otherwise. We estimate the following binomial GEE model [3] assuming an AR(1) working correlation structure (for the residuals of each pilot individual):

$$\Pr(y_{it} = 1 \mid D_{it}) = p_{it} = \frac{\exp(D_{it}\beta + \kappa_t)}{1 + \exp(D_{it}\beta + \kappa_t)},$$

where κ_t is a wave-specific offset that adjusts for secular trends in PrEP use or HIV testing observed in the RCS comparison group. Let π_t denote the estimated population proportion of the outcome in the RCS sample in wave t. The offset is calculated as

$$\kappa_t = \log\left(\frac{\pi_t}{1 - \pi_t}\right).$$

This offset ensures that the baseline log-odds in each wave match those observed in the comparison group. Thus, the parameter β captures the post-intervention shift in log-odds relative to the counterfactual trend established by the RCS comparison group. To ease interpretation, Figures 2 and 3 in the main text expresses this in terms of differences in predicted probabilities: we compute $Pr(y = 1 \mid D = 1) - Pr(y = 1 \mid D = 0)$, where these probabilities are computed for each wave using the wave-specifc offsets κ_t and then averaged across waves. Reported 95% confidence intervals are based on Huber-White standard errors.

A.2 Propensity score adjustment for RCS baseline covariates

As shown in Table 1 in the main text, there are substantial differences at baseline between the pilot and RCS population in terms of sociodemographic variables, PrEP knowledge and PrEP, use as well as sexual health-related behaviors. This raises the question to what extent the estimate from the pilot cohort is transportable to the "general" population as represented by the RCS. For a discussion of transportability, see [2, 5, 7, 1]. For a related application, see [6]. In the main text, we employ inverse odds of sampling reweighting [7] which rebalances the covariate distribution of the pilot sample towards that of the RCS at baseline.

Concretely, let $S = 1$ denote membership of the intervention sample and $S = 0$ denote membership for the RCS sample. Let X denote a vector of baseline covariates, which includes age at baseline, indicator variables for low and high income, unemployment, full-time employment, a

college degree, being bisexual, and living in Chengdu. In the extended specification we include condomless anal sex in the last three months, having ever heard of PrEP, having used PrEP in the last three months, or taken an HIV test in the last three months. We then estimate the propensity score for intervention-sample membership, $e(X)$,

$$e(X) = \Pr(S = 1 \mid X) = \frac{\exp(\delta_0 + \delta' X)}{1 + \exp(\delta_0 + \delta' X)}.$$

We use logistic regression in the combined intervention and RCS sample to obtain fitted values $\hat{e}(X_i)$ for each individual. The inverse-odds-of-sampling weight (IOSW) for individual i is given by

$$w_i = \begin{cases} \frac{1 - \hat{e}(X_i)}{\hat{e}(X_i)} \times \frac{\hat{\pi}}{1 - \hat{\pi}}, & \text{if } S_i = 1, \\ 0, & \text{if } S_i = 0, \end{cases}$$

where $\hat{\pi} = n_1/(n_0 + n1)$ is the sample proportion in the intervention group, with n_1 and n_0 denoting the intervention and RCS sample sizes, respectively. We then use w_i as observation weights when estimating the GEE models. This reweights the intervention sample to resemble the baseline RCS population in terms of characteristics X.

Sensitivity analyses As additional sensitivity analyses, we examine whether the results are robust to alternative approaches of addressing baseline differences between the pilot and RCS samples. First, we restrict the pilot sample to participants whose estimated sample-membership propensity scores fall within the common support of the RCS sample. Second, we use full matching on the propensity score [5, 4] in the combined baseline pilot-RCS sample to construct weights that improve baseline covariate balance before re-estimating the GEE models.

Table A1: Sensitivity analyses.

	PrEP use		HIV testing	
Analysis	Estimate	95% CI	Estimate	95% CI
Main text model[a]	19.9	(13.2, 26.6)	6.8	(0.9, 12.7)
Common support[b]	22.5	(17.5, 27.5)	6.3	(1.7, 10.8)
Full matching[c]	18.7	(11.1, 26.3)	6.7	(0.6, 12.8)

Note: Estimates are percentage-point changes from GEE models with RCS wave-specific outcome offsets. All three rows use the extended baseline covariate set: age, education, employment, income, bisexual identity, Chengdu residence, condomless anal sex, and PrEP awareness; the PrEP model additionally includes baseline HIV testing, while the HIV-testing model includes baseline PrEP use.
[a] Model with inverse odds of sampling weights. Used in main text.
[b] Common-support model. Limited to pilot participants with fitted propensity scores within the RCS 1st and 99th percentiles.
[c] Full matching model. Creates propensity-score subclasses with at least one pilot and one RCS baseline observation and weights each pilot participant by the RCS-to-pilot ratio in its subclass (weights are normalized to sum to the pilot baseline sample size).

Table A1 shows the resulting estimates (with estimates from the main model in the first row for reference). For the PrEP use outcome, the common support GEE estimate is slightly larger, while the full matching GEE estimate is slightly smaller. In all cases, the robust 95% confidence interval excludes zero. For the HIV testing outcome, the common support estimate as well as the

estimate obtained via full matching on the propensity score are very similar. Using full matching, the 95% confidence interval for HIV testing is slightly wider, but still clearly different from zero.

References

[1] Benjamin Ackerman, Catherine R. Lesko, Juned Siddique, Ryoko Susukida, and Elizabeth A. Stuart. Generalizing randomized trial findings to a target population using complex survey population data. *Statistics in Medicine*, 40(5):1101–1120, 2021.

[2] Stephen R. Cole and Elizabeth A. Stuart. Generalizing evidence from randomized clinical trials to target populations: The ACTG 320 trial. *American Journal of Epidemiology*, 172(1): 107–115, 2010.

[3] Kung-Yee Liang and Scott L. Zeger. Longitudinal data analysis using generalized linear models. *Biometrika*, 73(1):13–22, 1986.

[4] Elizabeth A. Stuart and Kerry M. Green. Using full matching to estimate causal effects in nonexperimental studies: Examining the relationship between adolescent marijuana use and adult outcomes. *Developmental Psychology*, 44(2):395–406, 2008.

[5] Elizabeth A. Stuart, Stephen R. Cole, Catherine P. Bradshaw, and Philip J. Leaf. The use of propensity scores to assess the generalizability of results from randomized trials. *Journal of the Royal Statistical Society: Series A*, 174(2):369–386, 2011.

[6] Kadija M. Tahlil, Audrey E. Pettifor, Jessie K. Edwards, Weiming Tang, Daniel Westreich, Titi Gbajabiamila, Hong Xian, Ucheoma Nwaozuru, Suzanne Day, Sonam J. Shah, Nora E. Rosenberg, David Oladele, Adesola Z. Musa, Lateef A. Blessing, Ponmile Ogunjemite, Donaldson F. Conserve, Temitope Ojo, Gbenga Ogedegbe, Oliver Ezechi, Juliet Iwelunmor, and Joseph D. Tucker. Uptake of facility-based HIV testing among adolescents and young adults in nigeria. *AIDS*, 40(2):239–248, 2026.

[7] Daniel Westreich, Jessie K. Edwards, Catherine R. Lesko, Elizabeth Stuart, and Stephen R. Cole. Transportability of trial results using inverse odds of sampling weights. *American Journal of Epidemiology*, 186(8):1010–1014, 2017.
