# Supplementary material for: Adding a Prevention Intervention to an Established App: The Blued+ HIV Prevention Pilot Study in Beijing and Chengdu, China
Source: J Int AIDS Soc. 2026 Sep 15;29(9):e70203. doi: 10.1002/jia2.70203 (PMC13575770; doi:10.1002/jia2.70203)
Supplement: Supplementary file 2 [file JIA2-29-e70203-s001.docx]

**Appendix: Modeling Strategy**

## **GEE model**

Let $y_{it}$ denote the observed binary outcome (PrEP use, HIV test) for individual $i$ ($i=1,\ldots,400$) in wave $t$ ($t=0,1,2$). $D_{it}$ is an indicator variable for the post-intervention period equal to 1 if $t\geq1$ and 0 otherwise.

We estimate the following binomial GEE model (Liang Zeger 1986) assuming an AR(1) working correlation structure:

$$Pr(y_{it}=1\mid D_{it})=p_{it}=\frac{exp(D_{it}\beta+\kappa_{t})}{1+exp(D_{it}\beta+\kappa_{t})},$$

where $\kappa_{t}$ is a wave-specific offset that adjusts for secular trends in PrEP use or HIV testing observed in the RCS comparison group. Let $\pi_{t}$ denote the estimated population proportion of the outcome in the RCS sample in wave $t$. The offset is calculated as

$$\kappa_{t}=log\left( \frac{\pi_{t}}{1-\pi_{t}} \right).$$

This offset ensures that the baseline log-odds in each wave match those observed in the comparison group. Thus, the parameter $\beta$ captures the post-intervention shift in log-odds relative to the counterfactual trend established by the RCS comparison group. To ease interpretation, Figures 2 and 3 in the main text expresses this in terms of differences in predicted probabilities: we compute $Pr(y=1\mid D=1)-Pr(y=1\mid D=0)$, where these probabilities are computed for each wave using the wave-specifc offsets $\kappa_{t}$ and then averaged across waves. Reported 95% confidence intervals are based on Huber-White standard errors.

## **Adjusting for RCS baseline covariates**

In our second specification, we adjust for differences in baseline covariates between the intervention group and the RCS comparison group using inverse odds of selection weights (Westreich et al 2017). Let $S=1$ denote membership of the intervention sample and $S=0$ denote membership for the RCS sample. Let $X$ denote a vector of baseline covariates, which includes age at baseline, indicator variables for low and high income, unemployment, full-time employment, a college degree, being bisexual, and living in Chengdu. We then estimate the probability of intervention sample membership,

$$e(X)=Pr(S=1\mid X)=\frac{exp(\delta_{0}+\delta^{'}X)}{1+exp(\delta_{0}+\delta^{'}X)}$$

via logistic regression in the combined intervention and RCS sample and obtain fitted values $\hat{e}(X_{i})$ for each individual.

The inverse-odds-of-sampling weight for individual $i$ is given by

$$w_{i}=\left\{ \begin{matrix} \frac{1-\hat{e}(X_{i})}{\hat{e}(X_{i})}\times\frac{\hat{\pi}}{1-\hat{\pi}}, & \text{ if }S_{i}=1, \\ 0, & \text{ if }S_{i}=0, \end{matrix} \right.$$

where $\hat{\pi}=n_{1}/(n_{0}+n1)$ is the sample proportion in the intervention group, with $n_{1}$ and $n_{0}$ denoting the intervention and RCS sample sizes, respectively. We then use $w_{i}$ as observation weights when estimating the GEE model. This reweights the intervention sample to resemble the baseline RCS population in terms of characteristics $X$.

The plot below compares raw standardized differences in means between the intervention and RCS sample in the left panel, and weighted standardized differences in means (using weights $w_{i}$) in the right panel. The weighted differences (right panel) are substantially closer to zero, indicating improved covariate balance after applying the inverse odds weights.

Westreich D, Edwards JK, Lesko CR, Stuart E, Cole SR. Transportability of Trial Results Using Inverse Odds of Sampling Weights. Am J Epidemiol. 2017 Oct 15;186(8):1010-1014. doi: 10.1093/aje/kwx164.
