# Supplementary material for: Adding a Prevention Intervention to an Established App: The Blued+ HIV Prevention Pilot Study in Beijing and Chengdu, China
Source: J Int AIDS Soc. 2026 Sep 15;29(9):e70203. doi: 10.1002/jia2.70203 (PMC13575770; doi:10.1002/jia2.70203)
Supplement: Supplementary file 3 [file JIA2-29-e70203-s003.docx]

Appendix. System Usability Scale

English version

Regarding the Blued+ app, how would you agree/disagree with the following statements?

|  | Strongly Agree (1) | Somewhat agree (2) | Neutral (3) | Somewhat disagree (4) | Strongly disagree (5) |
| --- | --- | --- | --- | --- | --- |
| 1. I think that I would like to use the Blued+ app frequently. |  |  |  |  |  |
| 2. I found the Blued+ app unnecessarily complex. |  |  |  |  |  |
| 3. I thought the Blued+ app was easy to use. |  |  |  |  |  |
| 4. I think that I would need the support of a technical person to be able to use the Blued+ app. |  |  |  |  |  |
| 5. I found that various functions in the Blued+ app were well integrated. |  |  |  |  |  |
| 6. I thought there was too much inconsistency in the Blued+ app. |  |  |  |  |  |
| 7. I would imagine that most people would learn to use the Blued+ app very quickly. |  |  |  |  |  |
| 8. I found the Blued+ app very cumbersome to use. |  |  |  |  |  |
| 9 I felt very confident using the Blued+ app. |  |  |  |  |  |
| 10. I needed to learn a lot of things before I could get going with the Blued+ app. |  |  |  |  |  |

Chinese version:

关于本项目应用程序，你是否同意/不同意以下陈述？

|  | 强烈同意 | 同意 | 中立 | 不同意 | 强烈不同意 |
| --- | --- | --- | --- | --- | --- |
| 我想我会经常使用 Blued+ |  |  |  |  |  |
| 我发现 Blued+ 过于复杂 |  |  |  |  |  |
| 我认为 Blued+ 使用起来很容易 |  |  |  |  |  |
| 我认为我需要技术人员的帮助才能使用 Blued+ |  |  |  |  |  |
| 我发现Blued+ 很好地集成了各种功能 |  |  |  |  |  |
| 我认为 Blued+ 中存在大量的不一致 |  |  |  |  |  |
| 我想大多数用户会很快学会使用Blued+ |  |  |  |  |  |
| 我发现 Blued+ 使用起来很别扭 |  |  |  |  |  |
| 我使用 Blued+ 时，感到很有信心 |  |  |  |  |  |
| 在使用 Blued+ 前，我需要学习很多知识 |  |  |  |  |  |
